# Mitochondrial complex I abnormalities is associated with tau and clinical symptoms in mild Alzheimer’s disease

**DOI:** 10.1186/s13024-021-00448-1

**Published:** 2021-04-26

**Authors:** Tatsuhiro Terada, Joseph Therriault, Min Su Peter Kang, Melissa Savard, Tharick Ali Pascoal, Firoza Lussier, Cecile Tissot, Yi-Ting Wang, Andrea Benedet, Takashi Matsudaira, Tomoyasu Bunai, Tomokazu Obi, Hideo Tsukada, Yasuomi Ouchi, Pedro Rosa-Neto

**Affiliations:** 1 Translational Neuroimaging Laboratory, McGill University Research Centre for Studies in Aging, Douglas Research Institute, Le Centre intégré universitaire de santé et de services sociaux (CIUSSS) de l’Ouest-de-l’Île-de-Montréal; Department of Neurology and Neurosurgery, Psychiatry and Pharmacology and Therapeutics, McGill University, 6875 Boulevard LaSalle, Montreal, H4H 1R3 Canada; 2 Department of Biofunctional Imaging, Preeminent Medical Photonics Education & Research Center, Hamamatsu University School of Medicine, 1-20-1 Handayama, Higashi-ku, Hamamatsu, 431-3192 Japan; 3 Department of Neurology, Shizuoka Institute of Epilepsy and Neurological Disorders, 886 Urushiyama, Aoi-ku, Shizuoka, 420-8688 Japan; 4 Central Research Laboratory, Hamamatsu Photonics KK, 5000 Hirakuchi, Hamakita-ku, Hamamatsu, 434-0041 Japan; 5 Hamamatsu PET Imaging Center, Hamamatsu Medical Photonics Foundation, 5000 Hirakuchi, Hamakita-ku, Hamamatsu, 434-0041 Japan

**Keywords:** Alzheimer’s disease (AD), Mitochondria, Tau, Amyloid, PET, [^18^F]BCPP-EF

## Abstract

**Background:**

Mitochondrial electron transport chain abnormalities have been reported in postmortem pathological specimens of Alzheimer’s disease (AD). However, it remains unclear how amyloid and tau are associated with mitochondrial dysfunction in vivo. The purpose of this study is to assess the local relationships between mitochondrial dysfunction and AD pathophysiology in mild AD using the novel mitochondrial complex I PET imaging agent [^18^F]BCPP-EF.

**Methods:**

Thirty-two amyloid and tau positive mild stage AD dementia patients (mean age ± SD: 71.1 ± 8.3 years) underwent a series of PET measurements with [^18^F]BCPP-EF mitochondrial function, [^11^C]PBB3 for tau deposition, and [^11^C] PiB for amyloid deposition. Age-matched normal control subjects were also recruited. Inter and intrasubject comparisons of levels of mitochondrial complex I activity, amyloid and tau deposition were performed.

**Results:**

The [^18^F]BCPP-EF uptake was significantly lower in the medial temporal area, highlighting the importance of the mitochondrial involvement in AD pathology. [^11^C]PBB3 uptake was greater in the temporo-parietal regions in AD. Region of interest analysis in the Braak stage I-II region showed significant negative correlation between [^18^F]BCPP-EF SUVR and [^11^C]PBB3 BP_ND_ (R = 0.2679, *p* = 0.04), but not [^11^C] PiB SUVR.

**Conclusions:**

Our results indicated that mitochondrial complex I is closely associated with tau load evaluated by [^11^C]PBB3, which might suffer in the presence of its off-target binding. The absence of association between mitochondrial complex I dysfunction with amyloid load suggests that mitochondrial dysfunction in the trans-entorhinal and entorhinal region is a reflection of neuronal injury occurring in the brain of mild AD.

**Supplementary Information:**

The online version contains supplementary material available at 10.1186/s13024-021-00448-1.

## Background

Alzheimer’s disease (AD) is characterized neuropathologically by the presence of senile plaques with extracellular aggregation of amyloid-β (Aβ) and tau neurofibrillary tangles [1]. In addition to Aβ and tau, neuroinflammation and oxidative stress reactions are observed in AD pathogenesis [2]. Mitochondria are responsible for not only energy supply of adenosine triphosphate (ATP) but also main intracellular source of reactive oxygen species (ROS) that cause cellular damage [2, 3]. Mitochondrial dysfunction was reported to be play a pivotal role in the pathogenesis in AD [4, 5]. Previous studies showed that Aβ directly affected mitochondrial bioenergetics and produced mitochondrial morphological change [6]. However, our recent study did not support the theory that Aβ deposition itself accelerates mitochondrial dysfunction in AD [7]. According to the amyloid cascade hypothesis of AD, Aβ is responsible for triggering downstream tau pathology which is closely related neurodegeneration [8]. Tau localizes predominantly in axons and contributes to the axonal transport [9]; the accumulation of pathological tau affects mitochondrial transport and causes mitochondrial dysfunction [5, 9]. Therefore, depicting mitochondrial dysfunction might be a useful marker to elucidate the effects of tau pathology on neuronal function. In turn, mitochondrial dysfunction leads to neuronal degeneration were reported to be associated with ROS [3, 10]. Oxidative stress contributes to tau phosphorylation and formation of neurofibrillary tangles [11]. Our recent work in tau transgenic mice (rTg4510 TauTg) showed a significance of tau pathology for mitochondrial dysfunction [12]. Thus, an in vivo study in the clinical setting is needed to verify the relationships between tau pathology and mitochondrial dysfunction in the living brains of patients with AD.

[^18^F]BCPP-EF is a newly developed PET tracer which binds to mitochondrial complex I (MC-I) [13]. In the electron transport chain (ETC) in mitochondria, MC-I is the first and rate-limiting enzyme required for ATP production and is a site of ROS production [14, 15]. [^18^F]BCPP-EF also permits the investigation of the topographical distribution of mitochondrial dysfunction, as well as relationships with tau-PET parametric changes. The aim of this study is to examine the relationship between MC-I availability, Aβ and tau deposition, and their influence on cognitive decline in mild AD patients using PET. We predict a pathophysiological association between tau pathology and MC-I availability in the clinical setting.

## Methods

### Participant

Thirty-two patients with relatively mild stage AD at the clinical dementia rating (CDR) of 0.5 or 1 (11 men and 21 women; mean age ± SD, 71.1 ± 8.3 years, all right-handed) were enrolled in this study [16]. Neuropsychological assessment for all patients comprised the Mental State Examination (MMSE) for global cognitive function. Twenty-five out of thirty-two patients completed Wechsler Memory Scale-Revised logical memory II (delayed recall) (story A and B) score (WMSR-LM) for episodic memory [17], and Frontal Assessment Battery (FAB) for executive function. The diagnosis of AD was based on the criteria of National Institute on Aging-Alzheimer’s Association workgroups on diagnostic guidelines for Alzheimer’s Disease (NIA/AA AD) [18]. To confirm the diagnosis of AD biologically, all patients underwent [^11^C] PiB PET scan to show the Aβ pathology as described below in detail. In addition, all patients underwent [^11^C]PBB3 scan or cerebrospinal fluid (CSF) test including total tau and phosphor-tau to confirm the tau pathology (described below). We confirmed that all patients showed positive [^11^C] PiB uptake in the cerebral cortex, and increased [^11^C]PBB3 uptake in the cerebral cortex or increased CSF phospho-tau. Furthermore, patients with cerebrovascular disease, white matter lesion (Fazekas score were 2 or 3) [19], hydrocephalus, brain tumor, epileptic foci, or traumatic brain injury were excluded based on brain MRI findings. Based on the electroencephalography, patients with epilepsy were excluded. In addition, participants whose Self-rating Depression Scale (SDS) scores were more than 60, indicating obvious depression, were excluded [20]. Patients with anxiety were excluded by using anxiety subscale of neuropsychiatric inventory (NPI) [21]. All patients with AD were taking a donepezil (5 mg) at entry.

Two groups of age-matched normal control subjects were also recruited. Control group for [^18^F]BCPP-EF evaluations consisted of 17 healthy subjects (10 men and 7 women: mean age ± SD, 66.6 ± 9.4 years) and control group for [^11^C]PBB3 evaluations consisted of 18 healthy subjects (8 men and 10 women: mean age ± SD, 69.5 ± 8.8 years). To reduce the radiation exposure in controls subjects, two control groups were investigated, and each control group underwent [^18^F]BCPP-EF or [^11^C] PBB3 respectively. All controls were right-handed and had no neurological problems, no history of head injury, psychiatric disease, serious medical illness, major surgery, or no family history of dementia. Their CDR score was zero, indicating no dementia [16]. Participants whose MMSE scores were less than 24, indicating global cognitive decline, were excluded [22]. Participants were excluded if they had MRI findings such as cerebrovascular disease, traumatic brain injury, brain tumor, hydrocephalus and epileptic foci. No significant differences in age and gender were found between the two control groups.

This study was reviewed and approved by the ethics committee of Shizuoka Institute of Epilepsy and Neurological Disorders (SIEND), Hamamatsu University School of Medicine, and Hamamatsu Medical Center. Written informed consent was obtained from all subjects to participate in the study.

### MRI scanning

Before the PET scan, 1.5 Tesla MRI (General Electric Healthcare, Japan) of the brain was performed with three-dimensional mode sampling to determine the brain areas in which to establish regions of interest (ROI). The scanning parameters were as follows: TR = 8.6 ms; TE = 3.6 ms; flip angle 25°; acquisition matrix = 256 × 224; FOV = 240 mm; 124 contiguous sagittal sections, each with a thickness of 1.4 mm; and a resolution of 0.9375 mm × 1.0714 mm × 1.4 mm. T2-weighted FLAIR images were also obtained to exclude the potential abnormalities described above. The MRI measurements and mobile PET gantry allows us to reconstruct PET images parallel to the intercommissural (anterior commissure-posterior commissure [AC-PC]) line without reslicing. Thus, we were able to locate the ROI in the target regions of the original PET images.

### PET measurement

All participants underwent a series of PET measurements using a high-resolution brain PET scanner (SHR12000; Hamamatsu Photonics K.K., Hamamatsu, Japan) [23], yielding 47 slices simultaneously. All thirty two AD patients completed [^18^F]BCPP-EF and [^11^C] PiB PET scan. Among the AD patients, sixteen out of thirty-two patients completed both [^18^F]BCPP-EF and [^11^C]PBB3 scan. A thermoplastic face mask was used to fix the head to the same place during the scans. Orbitomeatal line was defined by direct visual inspection of the subject and was aligned with a laser. On the other hand, the landmarks on the MR image were AC-PC line and orbitomeatal line. We analyzed the angles between the AC-PC line and orbitomeatal line on the MRI image. The PET gantry was set parallel to the AC-PC line determined by MRI by tilting and moving the gantry for each study. After backprojection and filtering (Hanning filter, cutoff frequency 0.2 cycles per pixel), the image resolution was 2.9 × 2.9 × 3.4 mm full-width half-maximum (FWHM). The voxel of each reconstructed image measured 1.3 × 1.3 × 3.4 mm. A 10-min transmission scan for attenuation correction with a ^68^Ge/^68^Ga source was conducted under resting conditions. After fasting overnight for at least 12 h, dynamic PET scans with 33 frames (serial emission scan: 6 frames× 10 s, 3 × 20, 6 × 60, 4 × 180, and 14 × 300) were obtained for 90 min, after a slow bolus venous injection (taking 1 min) of a 2 MBq/kg dose of [^18^F]BCPP-EF (106.8 ± 20.6 MBq). Within a few weeks allowance interval, dynamic PET scans with 29 frames (serial emission scan: 6 frames× 10 s, 3 × 20, 6 × 60, 4 × 180, and 10 × 300) were also obtained for 70 min after injection of 6 MBq/kg dose of [^11^C] PBB3 (295.9 ± 55.7 MBq). A few months prior to the current study, all patients were scanned with [^11^C] PiB PET to confirm the AD diagnosis biologically. After fasting overnight for at least 12 h, dynamic PET scans with 25 frames (serial emission scan: 6 frames × 10 s, 3 × 20, 2 × 60, 2 × 180, 8 × 300) were obtained for 70 min after injection of a 6 MBq/kg dose of [^11^C] PiB (255.1 ± 49.6 MBq). No arterial sampling was performed along with the series of PET measurements.

### PET imaging data processing

All PET data processing procedures were performed using PMOD 3.4 software (PMOD Technologies Ltd., Zurich, Switzerland). Co-registering the MRI and PET images was also determined by PMOD. To evaluate mitochondrial availability, semiquantitative evaluation based on the standardized uptake value ratio (SUVR) was calculated as reported recently [7]. The parametric [^18^F]BCPP-EF late-phase images were extracted from the interval between 70 and 90 min after injection. The standardized uptake value (SUV) of each region were divided by the SUV of whole brain as a reference region, and expressed as the SUVR image. Although a recent research demonstrated that centrum semiovale could be used as a reference region for estimating SUVR [17], our previous report showed that the semiquantitative value of [^18^F]BCPP-EF SUVR relative to the whole brain was positively correlated with the quantitatively estimated value of [^18^F]BCPP-EF volume of the distribution (V_T_) [7]. In addition, ROIs were manually drawn over the centrum semiovale on the MR image of each subject, these ROIs were placed onto corresponding original [^18^F]BCPP-EF SUVR parametric images. [^18^F]BCPP-EF SUVR image relative to the centrum semiovale was also created. We confirmed a better correlation between semiquantitative value of [^18^F]BCPP-EF SUVR relative to the whole brain and [^18^F]BCPP-EF SUVR relative to the centrum semiovale (Sup. Figure 1). Therefore, the use of [^18^F]BCPP-EF SUVR relative to the whole brain was adequate for semiquantitative evaluation.

To evaluate tau accumulation, the binding potential (BP_ND_) of [^11^C]PBB3 was estimated with the simplified reference tissue model (SRTM) as described elsewhere [23]. Irregular manual ROI were located on the pons on the MR image in reference to MRI atlas. These ROIs were then automatically transferred onto corresponding [^11^C]PBB3 parametric images. The pons ROI was selected as the reference region. A global [^11^C]PBB3 BP_ND_ outside the mean + 2SD was considered be abnormal.

To evaluate Aβ accumulation, we used SUVR, normalized by the cerebellar grey matter. MRI was also used to set the ROI of the cerebellum. A global [^11^C] PiB SUVR larger than 1.4 was considered to be abnormal [24].

After creating SUVR or BP_ND_ images, all PET images were normalized to the Montreal Neurological Institute (MNI) space and smoothed with an isotropic Gaussian kernel of 8 mm at FWHM. Then, all the image matrix and pixel resolution were interpolated to match those of the International Consortium for Brain Mapping (ICBM)-152 (193 × 229 × 193 with 1 mm pixel resolution).

Even though we used a high-resolution scanner and dedicated means for accurate positioning of PET and MRI images, PET data were corrected for partial volume using the Muller-Gartne method [19].

### Voxel-based analysis

To identify the brain region that exhibits decreased levels of [^18^F]BCPP-EF SUVR and higher levels of [^11^C]PBB3 BP_ND_ in the patients with AD than in age-matched control subjects, we performed a voxel-based t-test using the VoxelStats toolbox (https://github.com/sulantha2006/VoxelStats) running on MATLAB 8.5.0 (The MathWorks, Natick, MA, USA) [25, 26]. All voxel-based regression analyses were corrected for multiple comparisons using random field theory threshold with a cluster threshold at *p* < 0.001. This VoxelStats allows a comparison between age-matched groups, or conversely it cannot be applied when comparing groups with a significant age difference (https://github.com/sulantha2006/VoxelStats). In this study, albeit there was a wide age-range with a high SD between them, no significant difference of age between groups allowed to dismiss the procedure in treating age as a covariate.

### ROI analysis

We focused on medial temporal areas, a site of early tau accumulation [27–29]. We created FreeSurfer-derived ROI corresponding anatomically to the Braak staging regions of tau pathology as described elsewhere: Braak stage I-II (i.e., trans-entorhinal cortex, entorhinal cortex and hippocampus) [30–32]. White matter voxels were removed from the Braak stage ROI by using ICBM-152 Gy matter atlas (http://www.bic.mni.mcgill.ca/ServicesAtlases/ICBM152NLin2009) (Fig. 2a). We blurred Braak stage ROI to a FWHM of 8 mm (the same resolution as the PET images). This ROI was then automatically transferred onto corresponding [^18^F]BCPP-EF SUVR, [^11^C]PBB3 BP_ND_, [^11^C] PiB SUVR images. Then, regional [^18^F]BCPP-EF SUVR, [^11^C]PBB3 BP_ND_, and [^11^C] PiB SUVR derived from this regional Braak ROI were determined. Using segmented MRI information allowed us to exclude data of the cerebrospinal fluid space. Pearson’s correlation analysis and linear regression analysis with the GraphPad Prism 5.0 package (GraphPad, San Diego, CA, USA) were calculated to explore the relationships among mitochondrial activity ([^18^F]BCPP-EF), tau deposition ([^11^C]PBB3), Aβ deposition ([^11^C]PiB), and neuropsychological tests (MMSE, WMSR-LM, and FAB). In addition, t-test was conducted to compare the mitochondrial activity or tau deposition between AD and control subjects. Statistical significance in the Braak I-II ROI was set to *p* < 0.05 without multiple comparison. Because this test was exploratory with a priori knowledge of tau induced mitochondrial dysfunction in AD [12], the region including the trans-entorhinal, entorhinal and hippocampal cortices was a central target of interest in this study.

Similarly, to reject the possibility for significant contribution of tau aggregated at the Braak III-VI stage, Pearson’s correlation analysis and t-test were conducted in the Braak stage III-IV region that covers the amygdala, parahippocampus, fusiform, lingual gyrus, insula, inferior temporal, lateral temporal, posterior cingulate, and inferior parietal cortex (Fig. 3a) and Braak stage V-VI region that covers the orbitofrontal, superior temporal, inferior frontal, cuneus, anterior cingulate, supramarginal gyrus, lateral occipital, precuneus, superior parietal, superior frontal, rostro medial frontal, paracentral, postcentral, precentral, and pericalcarine (Fig. 4a).

## Results

### Demographic and clinical characteristics

Demographic and AD-related clinical factors are presented in Table 1.

**Table 1:** Demographic and clinical characteristics of the Alzheimer’s disease patient group and control groups

	Alzheimer’s disease		Normal Control		Normal range
	Total (*n* = 32)	Subgroup for analysis between [^18^F] BCPP and [^11^C]PBB3 PET (*n* = 16)	[^18^F] BCPP PET (*n* = 17)	[^11^C]PBB3 PET (*n* = 18)	
Age (years)	71.1 ± 8.3	71.3 ± 5.4	66.6 ± 9.4	69.5 ± 8.8	
Men/women (number)	11/21	4/12	10/7	8/10	
Disease duration (years)	2.8 ± 1.1	2.4 ± 0.8			
Clinical Dementia Rating (0/0.5/1)	0/20/12	0/12/4	17/0/0	18/0/0	
Mini-Mental State Examination (/30)	22.2 ± 4.1	22.8 ± 3.7	27.5 ± 1.9	27.4 ± 1.9	> 23
Wechsler Memory Scale-Revise logical memory II (delayed recall) (story A and B) (/50)	5.4 ± 5.3	6.8 ± 5.8			> 11
Frontal Assessment Battery (/18)	10.1 ± 2.6	10.7 ± 2.0			
Self-rating Depression Scale (/100)	36.9 ± 8.6	37.6 ± 8.3			< 60
Regional [^18^F] BCPP-EF standardized uptake value ratio (Braak I-II / III-IV/ V-VI ROI)	0.59 ± 0.06/ 0.85 ± 0.05/ 0.89 ± 0.04	0.61 ± 0.04/ 0.84 ± 0.03/ 0.88 ± 0.03	0.63 ± 0.03/0.89 ± 0.02/0.93 ± 0.03		
Regional [^11^C]PBB3 PET binding potential (Braak I-II / III-IV/ V-VI ROI)	0.25 ± 0.08/ 0.38 ± 0.13/ 0.31 ± 0.11	0.25 ± 0.08/ 0.38 ± 0.13/ 0.31 ± 0.11		0.16 ± 0.07/ 0.22 ± 0.09/ 0.17 ± 0.08	
Regional [^11^C] PiB PET standardized uptake value ratio (Braak I-II / III-IV/ V-VI ROI)	1.31 ± 0.17/ 1.69 ± 0.24/ 1.78 ± 0.25	1.39 ± 0.17/ 1.67 ± 0.25/ 1.78 ± 0.24			
[^11^C] PiB PET global cortical standardized uptake value ratio	1.88 ± 0.24	1.89 ± 0.21			< 1.4
[^11^C]PBB3 PET global cortical binding potential	0.38 ± 0.11	0.38 ± 0.11		0.17 ± 0.05	
Cerebrospinal fluid					
Total tau (pg/ml)	712.1 ± 207.2				< 1200
Phospho-tau (pg/ml)	86.7 ± 26.1				< 50

Data are presented as mean ± SD (range)

To confirm tau pathology, sixteen out of thirty-two patients completed [^11^C]PBB3 PET scan. The rest of sixteen patients underwent cerebrospinal fluid tests

### Comparison of the [^18^F]BCPP-EF SUVR, and [^11^C]PBB3 BP_ND_, between AD and control groups

**Fig. 1:**
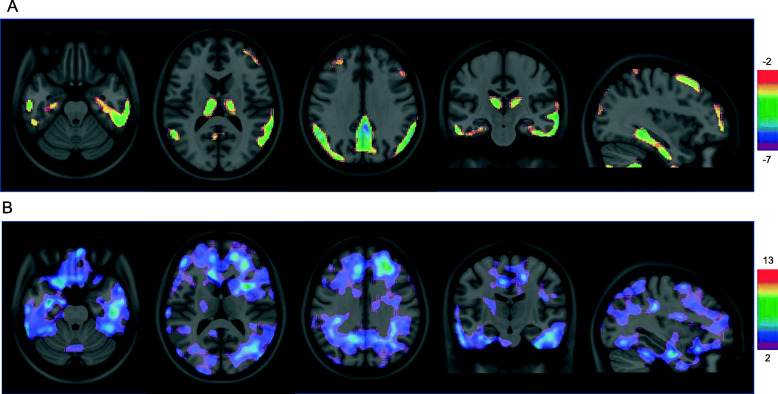
Voxelwise results for [^18^F]BCPP-EF and [^11^C]PBB3 in the whole brain in AD patients. T statistical parametric map corrected for multiple comparisons using random field theory at *p* < 0.001, overlaid on the Montreal Neurological Institute 152 reference template. Color bar represents the T-value. **a** The regions in which the [^18^F]BCPP-EF SUVR in the AD group were significant reduced compared to control group (**b**) The regions in which the [^11^C]PBB3 BP_ND_ in the AD group were significant increased compared to control group

A voxel-based t-test showed that a significant reduction in [^18^F]BCPP-EF SUVR was observed in the medial and lateral temporal, parietal, frontal cortex, precuneus, cingulate gyrus, thalamus, and basal ganglia in the total AD group compared with the control group (Fig. 1a). A voxel-based regression model showed that a significant increase in [^11^C]PBB3 BP_ND_ was observed in the medial and lateral sides of the temporal and parietal lobes including the parahippocampus and hippocampus in the AD group compared with the control group (Fig. 1b).

### ROI analysis

**Fig. 2:**
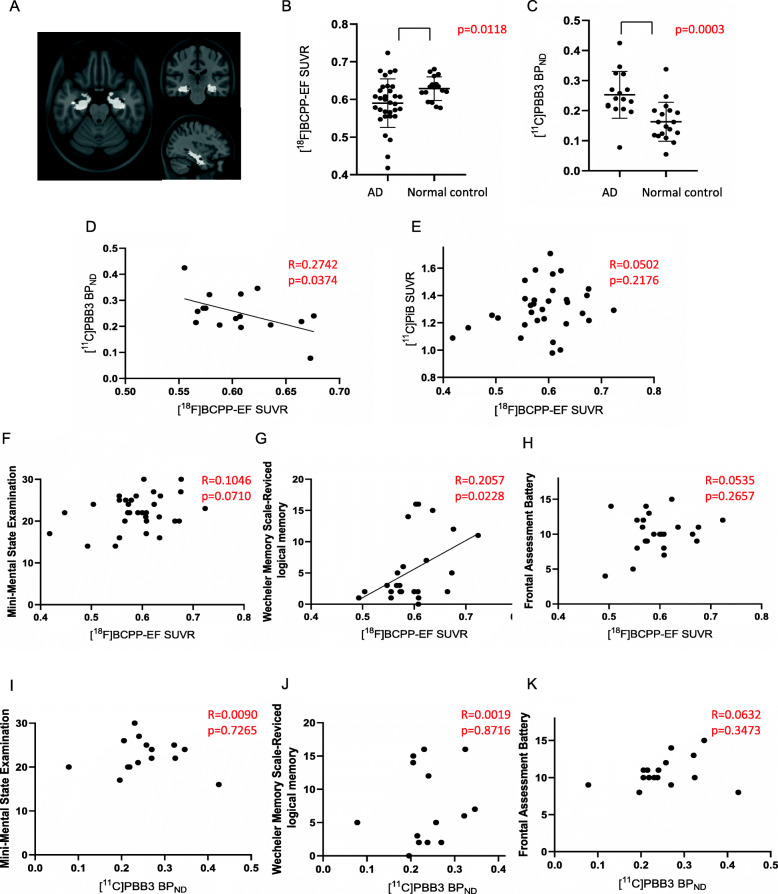
Correlation analyses between [^18^F]BCPP-EF SUVR and [^11^C]PBB3 BP_ND_, [^11^C] PiB SUVR, or neuropsychological battery scores in the Braak stage I-II region of interest (ROI) in patients with AD. **a** Brain area of Braak stage I-II ROI overlaid on the Montreal Neurological Institute 152 reference template. **b** The regional [^18^F]BCPP-EF SUVR in AD (*n* = 32) was significant lower than that in the controls (*n* = 17). **c** The regional [^11^C]PBB3 BP_ND_ in AD (n = 32) was significant higher than that in the controls (*n* = 18). **d** There was significant negative correlation of [^18^F] BCPP-EF SUVR with [^11^C]PBB3 BP_ND_ (*n* = 16)_._ **e** Correlation of [^18^F]BCPP-EF SUVR with [^11^C] PiB SUVR failed to reach significance (n = 32). **f**, **g**, **h** There was significant positive correlation of [^18^F] BCPP-EF SUVR with Wechsler Memory Scale-Revised logical memory score (*n* = 25), but not Mini-Mental Examination score (n = 32) or Frontal Assessment Battery score (n = 25). **i**, **j**, **k** There was no significant positive correlation of [^11^C]PBB3 BP_ND_ with Mini-Mental Examination score, Wechsler Memory Scale-Revised logical memory score, or Frontal Assessment Battery score (n = 16)

**Fig. 3:**
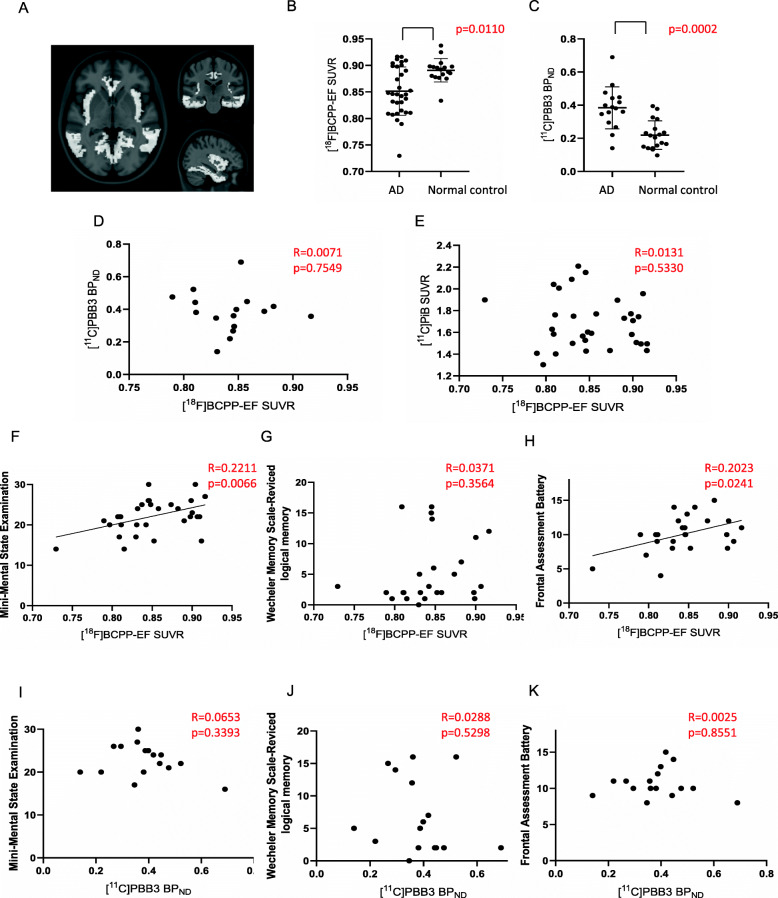
Correlation analyses between [^18^F]BCPP-EF SUVR and [^11^C]PBB3 BP_ND_, [^11^C] PiB SUVR, or neuropsychological battery scores in the Braak stage III-IV region of interest (ROI) in patients with AD. **a** Brain area of Braak stage III-IV ROI overlaid on the Montreal Neurological Institute 152 reference template. **b** The regional [^18^F]BCPP-EF SUVR in AD (*n* = 32) was significant lower than that in the controls (*n* = 17). **c** The regional [^11^C]PBB3 BP_ND_ in AD (n = 32) was significant higher than that in the controls (*n* = 18). **d**, **e** There was no significant negative correlation of [^18^F] BCPP-EF SUVR with [^11^C]PBB3 BP_ND_ (*n* = 16) or [^11^C] PiB SUVR (*n* = 32). **f**, **g**, **h** There was significant positive correlation of [^18^F] BCPP-EF SUVR with Mini-Mental Examination score (*n* = 32) or Frontal Assessment Battery score (*n* = 25), but not Wechsler Memory Scale-Revised logical memory score (*n* = 25). **i**, **j**, **k** There was no significant positive correlation of [^11^C]PBB3 BP_ND_ with Mini-Mental Examination score, Wechsler Memory Scale-Revised logical memory score, or Frontal Assessment Battery score (*n* = 16)

**Fig. 4:**
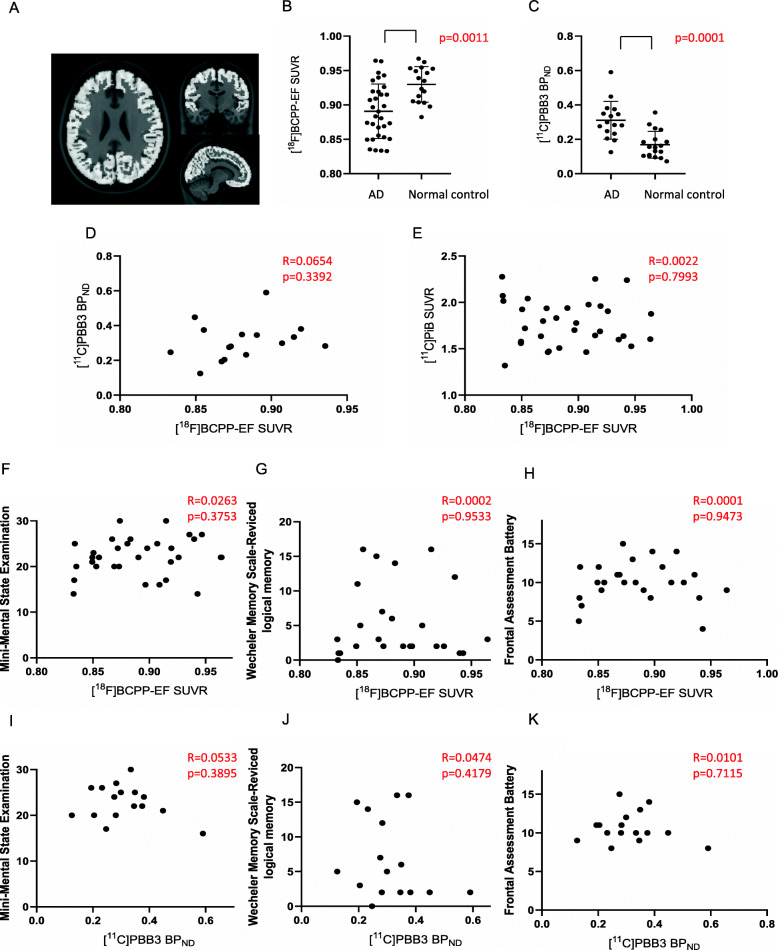
Correlation analyses between [^18^F]BCPP-EF SUVR and [^11^C]PBB3 BP_ND_, [^11^C] PiB SUVR, or neuropsychological battery scores in the Braak stage V-VI region of interest (ROI) in patients with AD. **a** Brain area of Braak stage V-VI ROI overlaid on the Montreal Neurological Institute 152 reference template. **b** The regional [^18^F]BCPP-EF SUVR in AD (n = 32) was significant lower than that in the controls (n = 17). **c** The regional [^11^C]PBB3 BP_ND_ in AD (n = 32) was significant higher than that in the controls (n = 18). **d**, **e** There was no significant negative correlation of [^18^F] BCPP-EF SUVR with [^11^C]PBB3 BP_ND_ (n = 16) or [^11^C] PiB SUVR (n = 32). **f**, **g**, **h** There was no significant positive correlation of [^18^F] BCPP-EF SUVR with Mini-Mental Examination score (n = 32), Wechsler Memory Scale-Revised logical memory score (n = 25), or Frontal Assessment Battery score (n = 25). **i**, **j**, **k** There was no significant positive correlation of [^11^C]PBB3 BP_ND_ with Mini-Mental Examination score, Wechsler Memory Scale-Revised logical memory score, or Frontal Assessment Battery score (n = 16)

Direct comparisons between [^18^F]BCPP-EF SUVR and [^11^C]PBB3 BP_ND_ identified a significant negative correlation in the Braak stage I-II ROI (Fig. 2a, d). However, there was no correlation between [^18^F] BCPP-EF SUVR and [^11^C] PiB SUVR (Fig. 2e). In addition, there were no significant correlations between [^18^F]BCPP-EF SUVR and [^11^C]PBB3 BP_ND_ or [^11^C] PiB SUVR in the Braak stage III-IV ROI and V-VI ROI (Figs. 3d, e, 4d, e).

There were positive correlations between the [^18^F]BCPP-EF SUVR and WMSR-LM score in the Braak stage I-II ROI (Fig. 2g), but not MMSE or FAB score (Fig. 2f, h). However, there were positive correlations between the [^18^F]BCPP-EF SUVR and MMSE score or FAB score in the Braak stage III-IV ROI (Fig. 3f, h), but not WMSR-LM score (Fig. 3g). There was no positive correlation between [^18^F]BCPP-EF SUVR and neuropsychological battery score in the Braak stage V-VI ROI (Fig. 4f, g, h). There was no positive correlation between [^11^C]PBB3 BP_ND_ and neuropsychological battery score in the Braak stage I-II, III-IV, V-VI ROI (Figs. 2i, j, k, 3i, j, k, 4i, j, k).

The regional [^18^F]BCPP-EF SUVR in the Braak stage I-II, III-IV, and V-VI ROI was significant lower in AD than in the controls (Figs. 2b, 3b, 4b). Between the controls and AD group without two patients who presented lower [^18^F]BCPP-EF SUVR in the Braak stage I-II ROI, the statistical significance was still observed (*p* = 0.0235) (data not shown). The regional [^11^C]PBB3 BP_ND_ in the Braak stage I-II, III-IV, and V-VI ROI was significant higher in AD than in the controls (Figs. 2c, 3c, 4c).

## Discussion

The aim of this study is to investigate the relationship of mitochondrial activity with tau pathology and its influence on clinical symptoms in mild AD dementia. The present study demonstrated decreased levels of MC-I availability in the temporal, parietal and frontal cortices, and significant increased level of tau load in the temporo-parietal and frontal cortices. Within the trans-entorhinal, entorhinal and hippocampal cortices (Braak stage I-II), levels of mitochondrial availability displayed a negative association with levels of tau aggregation, suggesting that intracellular and partly extracellular aggregation of tau is an important detrimental event for neuronal failure caused by MC-I availability in AD. No correlation was observed between levels of MC-I availability and Aβ accumulation. Altered MC-I availability in the trans-entorhinal and entorhinal region reflects a loss of normal homeostasis in neurons as an early biomarker of pathophysiology in AD.

It is increasingly recognized that mitochondrial dysfunction occurs in earlier disease stages [7, 10, 23] in AD. The present finding of mutual relation between mitochondrial dysfunction and tau pathology in AD is consistent with previous reports [7, 23], suggesting the coexistence of mitochondrial dysfunction and tau pathology. Because of this study’s cross-sectional design, it was not possible to infer whether mitochondrial dysfunction or these abnormal protein depositions occurs first. Since these analyses were exploratory, further study with multiple comparison will be needed. However, when the MC-I availability as measured with [^18^F]BCPP-EF SUVR is plotted against tau load measured with [^11^C]PBB3 BP_ND_, the results provided the significant negative correlation in the ROI of Braak stage I-II. In addition, we confirmed altered MC-I availability and tau pathology in the Braak stage I-II ROI in AD, although the standard deviation was relatively large. According to the amyloid-β cascade hypothesis [33, 34], Aβ triggers tau pathology which is accompanied by subsequent neurodegeneration [8]. According to the Braak’s stage of tau distribution, pathological tau deposition initially occurs in the medial temporal area, followed by the frontal and lateral temporal area, and later spread to the primary motor and sensory areas [28, 33]. Tau pathology sequentially induces neurodegeneration, and greater tau load is related to longer disease duration [33].

Medial temporal area covering trans-entorhinal and entorhinal cortex are particularly vulnerable to AD-related pathological changes such as tau accumulation [28, 35]. Thus, it is likely that tau aggregation in AD constitutes an important detrimental event, imposing energy failure in neurons and neurodegeneration. Negligible associations between [^18^F]BCPP-EF with [^11^C] PiB suggested that mitochondrial damage in the trans-entorhinal and entorhinal regions is not directly linked directly to Aβ deposition in mild AD. In addition, the absence of association between reduced [^18^F]BCPP-EF with increased [^11^C] PiB may indicate that MC-I dysfunction is late and not early in the AD disease process. However, it is important to note that [^11^C] PiB SUVR is not a strong correlate of Aβ oligomers, toxic Aβ species and it is plausible that no correlation between MC-I uptake and the reactive or “benign” accumulation of Aβ plaques that happen with aging and are accelerated in AD.

[^18^F]BCPP-EF SUVR in Braak stage I-II area covering trans-entorhinal cortex and entorhinal cortex was positively correlated with the WMSR-LM score that reflects logical memory function [36]. Memory impairments are referred to as the early clinical manifestation of AD [37]. Correspondingly, in vivo imaging showed that tau pathology appears early in the medial temporal lobe, with medial temporal lobe atrophy and memory impairments continuing in AD [28]. Our result of reduced mitochondrial activity correlating with memory decline gives an additional important message such as a considerable contribution of an early mitochondrial dysfunction (supporting a mitochondrial cascade hypothesis [4]) to this tau-based memory impairment. In contrast, we failed to show any relationship between [^18^F]BCPP-EF SUVR in Braak stage I-II area and MMSE or FAB score. On the other hand, [^18^F]BCPP-EF SUVR in Braak stage III-IV area covering limbic area including temporal cortex and insula was positively correlated with the MMSE or FAB score. The MMSE and FAB scores reflect the levels of general cognition [22] and frontal lobe-based executive function [38]. These findings are consistent with the present clinical evaluation that the AD patients examined in this study were rated as those with mild severity of dementia. The lack of correlation between MC-I availability and MMSE in Braak stage I-II area might be due to the fact that this correlation builds up with the spreading of the pathological process to the later stages of tau accumulation in Braak stages III-IV area. In addition, the correlation between MC-I availability and FAB in not Braak stage I-II area but Braak stages III-IV area support the notion that executive dysfunction in AD appears after memory problems [37]. Thus, the level of [^18^F]BCPP-EF binding in the trans-entorhinal cortex and entorhinal cortex can be a biomarker that predicts the memory-dominant cognitive decline in mild AD. Although our previous study indicated the association between tau deposition detected by [^11^C]PBB3 BP_ND_ and MMSE score in the whole brain analysis [23], our ROI analysis failed to show any relationship between [^11^C]PBB3 BP_ND_ and neuropsychological battery scores in Braak stages area. This discrepancy might be a due to methodological differences. The relatively large size of the ROI (especially at the high Braak stages) used in this study might be an explanation for the lack of correlation.

The correlation between mitochondrial dysfunction and tau deposition was restricted to the early sites of tau accumulation, i.e. medial temporal region. In this study, patients with mild AD (CDR0.5 or 1) were recruited. Therefore, there might be a chance of positive correlation between mitochondrial dysfunction and tau deposition more widely if preclinical or more severe stage of AD were recruited. Other than the genetical reason, mitochondrial dysfunction can be caused by various factors such as neuroinflammation and misfolded proteins like Aβ and tau [4, 34]. Once mitochondria are impaired, mitochondria-generated ROS causes neuroinflammation [2, 34, 39], and mitochondria are in turn targeted at by generated ROS [2, 40, 41]. Our results indicated the presence of MC-I dysfunction in the subcortical areas outside the cortical region. Subcortical area such as thalamus and basal ganglia were reported to be one of the brain areas showing mitochondrial dysfunction, which were associated with wide neuronal loss and synaptic alternations [42]. Future studies are needed to know how neuroinflammation contributes to the exacerbation of the brain milieu in a vicious cycle by tau aggregation and mitochondrial dysfunction [34].

There are several limitations that must be considered when interpreting this study. The sample size was small. Although there was no significant difference of age between AD patients and controls subjects, the relatively wide age range and high standard deviation of both groups might be a confounding factor. Hence, it might be more appropriate to consider many factors affecting the brain functions and morphology such as age, education, diet etc. as confounding covariates. Although the setting of ROIs was completely automatic, any automatic manipulation would suffer more or less misplacement of ROIs even in spatially-normalized images (which sometimes accompanies distortion), which might cause an interpretation bias in this study. However, this possible bias is a universal problem to be overcome in in vivo imaging studies even using spatially normalized images. To reduce the criticism, we are referring to the widely-accepted atlas. Previous studies indicated that the first-generation of tau tracers such as [^11^C]PBB3 showed off-target binding in the basal ganglia, thalamus, choroid plexus, and venous sinus [43]. Therefore, we need to be cautious about describing any increase in [^11^C]PBB3 BP_ND_ in these areas. The spill-over radioactivity might affect the signal. The localization of the choroid plexus in the lateral ventricles, in close proximity to the hippocampus could lead to problematic quantification of the tracer retention and asymmetric fixation of [^11^C]PBB3 [44]. Additional studies with other tau PET tracer are needed to confirm our result [45]. In addition, an association between mitochondrial dysfunction and tau deposition could only be examined in AD patients at relatively mild clinical stage, and the utility of MC-I PET in predementia AD is not known. Despite this preliminary condition, the present information about within subject changes in these pathophysiological biomarkers would help understand the prognostic use of mitochondrial dysfunction as a biomarker in mild AD. To confirm this, further studies with large sample size and several disease stages of AD patients are needed by focusing changes in the associations between mitochondrial dysfunction and misfolded proteins aggregation or neuronal damage.

## Conclusions

This study provides in vivo evidence that MC-I impairment coincides with tau deposition in the trans-entorhinal and entorhinal region, which contributes to the memory decline in mild AD. Assessing MC-I availability by [^18^F]BCPP-EF PET may constitute a valuable tool for evaluating the pathophysiology of AD from the perspective of mitochondrial hypothesis, which can open numerous therapeutic avenues.

## Supplementary Information

**Additional file 1 **: **Supplemental Figure 1**. There was significant positive correlation between the semiquantitative value of [^18^F]BCPP-EF SUVR relative to the whole brain and [^18^F]BCPP-EF SUVR relative to the centrum semiovale in the average of the all ROIs (manually located bilaterally in the cerebellum, anterior cingulated cortex, caudate, putamen, thalamus, posterior cingulated cortex, precuneus, superior frontal cortex, middle frontal cortex, occipital cortex, lateral temporal cortex, medial temporal cortex including hippocampus, parahippocampus, amygdala, parietal cortex, pons, and midbrain).

## Data Availability

Anonymized data not published within the article will be shared on reasonable request from any qualified investigator.\

## Abbreviations

AD: Alzheimer’s disease
Aβ: Amyloid-β
ATP: Adenosine triphosphate
ROS: Reactive oxygen species
BCPP-EF: 2-tert- butyl-4-chloro-5–2Hpyridazin-3-one
PET: Positron emission tomography
MC-I: Mitochondrial complex I
ETC: Electron transport chain
CDR: Clinical dementia rating
MMSE: Mental State Examination
WMSR-LM: Wechsler Memory Scale-Revised logical memory
FAB: Frontal Assessment Battery
SDS: Self-rating Depression Scale
NPI: Neuropsychiatric inventory
NIA/AA AD: National Institute on Aging-Alzheimer’s Association workgroups on diagnostic guidelines for Alzheimer’s Disease
PiB: Pittsburg compound B
PBB3: Pyridinyl-butadienyl-benzothiazole 3
CSF: Cerebrospinal fluid
MRI: Magnetic resonance imaging
ROI: Regions of interest
AC-PC: Anterior commissure-posterior commissure
FWHM: Full-width half-maximum
SUVR: Standardized uptake value ratio
SUV: Standardized uptake value
BP_ND_: Binding potential
SRTM: Simplified reference tissue model
MNI: Montreal Neurological Institute
ICBM: International Consortium for Brain Mapping
