# Supplementary figures and images for: Mitochondrial complex I abnormalities is associated with tau and clinical symptoms in mild Alzheimer’s disease
Source: Mol Neurodegener. 2021 Apr 26;16:28. doi: 10.1186/s13024-021-00448-1 (PMC8074456; doi:10.1186/s13024-021-00448-1)

## Slide 1
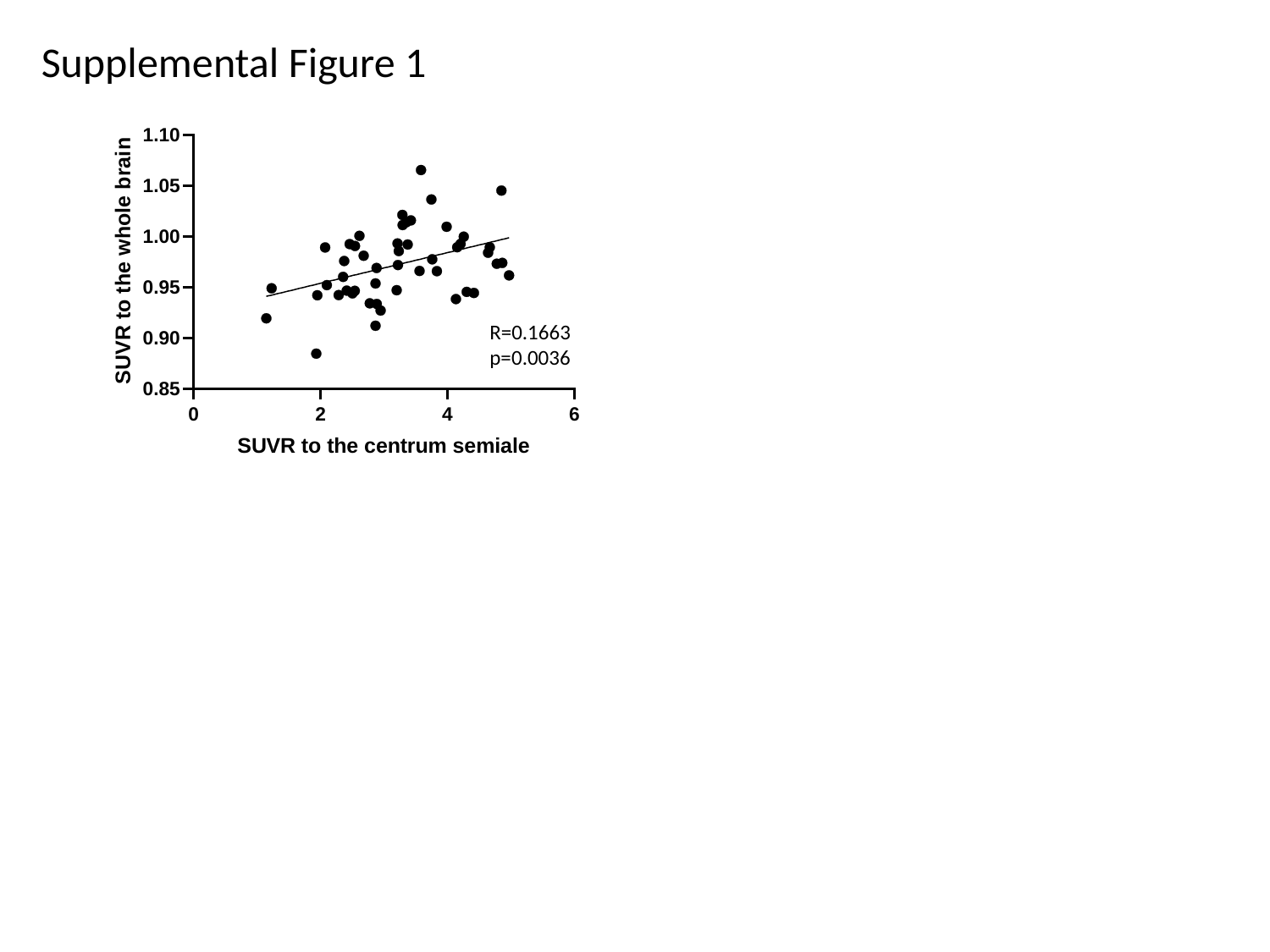

Supplemental Figure 1
R=0.1663
p=0.0036

Supplement: Supplementary file 1 — Additional file 1 : Supplemental Figure 1. There was significant positive correlation between the semiquantitative value of [18F]BCPP-EF SUVR relative to the whole brain and [18F]BCPP-EF SUVR relative to the centrum semiovale in the average of the all ROIs (manually located bilaterally in the cerebellum, anterior cingulated cortex, caudate, putamen, thalamus, posterior cingulated cortex, precuneus, superior frontal cortex, middle frontal cortex, occipital cortex, lateral temporal cortex, medial temporal cortex including hippocampus, parahippocampus, amygdala, parietal cortex, pons, and midbrain). [file 13024_2021_448_MOESM1_ESM.ppt]
